# International Developmental Pediatrics Association: Global equity in child development and disability

**DOI:** 10.1136/bmjpo-2026-004769

**Published:** 2026-08-27

**Authors:** Ezgi Ozalp Akin, Revan Mustafayev, Roopa Srinivasan, Koyeli Sengupta, Ma. Rochelle Pacifico, Lama Charafeddine, Kirsten Ann Donald, Tarana Seyid Mammadova

**Affiliations:** 1 Department of Pediatrics, Developmental Pediatrics Division, Ankara University School of Medicine, Ankara, Ankara, Turkey; 2 International Developmental Pediatrics Association; 3 Ummeed Child Development Center, Mumbai, Maharashtra, India; 4 Developmental Pediatrics, Ummeed Child Development Center, Mumbai, Maharashtra, India; 5 Developmental Pediatrics, De La Salle Medical and Health Sciences Institute, Dasmariñas, Calabarzon, Philippines; 6 American University of Beirut Faculty of Medicine, Beirut, Lebanon; 7 Divison of Developmental Paediatrics, University of Cape Town Department of Paediatrics and Child Health, Rondebosch, South Africa, South Africa; 8 Children’s Rehabilitation Center, Republican Pediatric Center, Baku, Azerbaijan

**Keywords:** Child, Low and Middle Income Countries, Rehabilitation, Child Health, Developing Countries

 The majority of the world’s child population and children with disabilities reside in low-income and middle-income countries (LMICs)^1^ where resources to support child development and children with disabilities are often scarce, fragmented or entirely unavailable. Professionals working in developmental paediatrics and allied disciplines in LMICs similarly face restricted access to training, mentorship, research and partnership opportunities.^2^ Even when there are such opportunities, they may be affected by longstanding hierarchies between high-income countries (HICs) and LMICs and inequities embedded within global health and academic systems.^3^

The International Developmental Pediatrics Association (IDPA) was established in 2015 during the first International Developmental Pediatrics Congress in Istanbul, Turkey, under the leadership of founding president Ilgi Ertem, together with Vibha Krishnamurthy (India), Mphele Mulaudzi (South Africa), Alexis Reyes (Philippines), Ghassan Issa (Lebanon), Ricardo Halpern (Brazil) and Nenad Rudic (Serbia) to address the inequities mentioned above.^2 4^ The IDPA’s vision is to achieve equity in developmental paediatrics and related services for children and families worldwide. Its mission is to create a scientific international platform to connect and support clinicians, researchers and policymakers working in the field of developmental paediatrics and allied disciplines globally to address the inequity in how children are supported to develop to their full potential. Its core values are as follows: (a) science and partnership for equity; (b) holistic, family-centred, strengths-based, community-oriented, transdisciplinary capacity building; (c) United Nations Convention on the Rights of the Child; (d) United Nations Convention on the Rights of Persons with Disabilities. Specific goals are to (a) provide an organisational home for professionals, (b) support research, documentation and writing, (c) develop South to South cooperation/collaboration, (d) promote partnership between professionals, families and communities, (e) enhance state of the art, evidence-based applicable models, programmes and policies, (f) promote leaders in countries, (g) support training and education, (h) support the foundation of Developmental Paediatrics and (i) enhance interdisciplinary collaborations.

Driven by this vision, mission, core values and goals, IDPA over the past 11 years organised six IDPA congresses hosted exclusively in LMICs as a deliberate strategy to strengthen local leadership, visibility and participation. These congresses, as flagship activities of IDPA, have created opportunities for clinicians, researchers, trainees, policy makers, self-advocates and families from across the world to exchange evidence, experiences and innovations while building networks of mentorship with a centre of gravity in the geographies where the need is greatest. Importantly, the congresses adopted explicit equity-oriented policies, including adherence to the WHO International Code of Marketing of Breastmilk Substitutes, refusal of funding from pharmaceutical and formula industries and prioritisation of scientific contributions originating from LMICs. Abstracts from HICs were accepted primarily when focused on marginalised populations such as refugees or conducted in LMICs in partnership with researchers from LMICs. In addition to ‘research’, submissions under a ‘model/programme’ category were also encouraged to provide space for the experiences, practices and perspectives of professionals who often lacked access to research funding, opportunities or international publication pathways, yet held critical knowledge and innovative approaches relevant to child development and disability. Collectively, these practices sought to challenge traditional inequities within global academic spaces, amplify voices that are often underrepresented and engage professionals directly with internationally recognised leaders in the field, receive mentorship and build collaborative networks.

Each of the six IDPA Congresses has addressed contemporary priorities in child development, disability and equity. To symbolically express the alignment of IDPA’s actions with articulated vision and mission, the congresses are deliberately held in early December on the dates that include the International Day for People with Disabilities. Over the span of six congresses, participants representing 79 countries have convened for a scientific discourse on themes represented by global and local priority topics in the field of developmental paediatrics and allied disciplines (table 1). The ‘Milton and Marjorie Lozoff, Mentoring and Mingling Dinner (M&M Dinner)’ held at every IDPA Congress has consistently provided opportunities for informal interactions between young professionals and globally recognised scientists and mentors in an informal and supportive round table dinner environment facilitating networking, exchange of ideas and the development of professional relationships. Each congress was conducted in a mutually beneficial partnership with local professional or non-governmental organisations that also aimed at facilitating the legitimacy of the regional and national advocacy efforts of the host organisation. As a result, local professionals from regions rarely represented in global congresses were able to come together, exchange perspectives and strengthen a more equitable global developmental paediatrics community.

**Table 1:** International Developmental Pediatrics Association Congresses

Congress	Location	Theme	Key highlights
First International Developmental Pediatrics Congress (2015)	Istanbul, Turkey	Addressing Early Childhood Development and Disability	Hosted by Ankara University 573 participants from 57 countries Promoted monitoring instead of screening child development Led to establishment of IDPA
Second IDPA Congress (2017)	Mumbai, India	A World of Difference	Hosted by Ummeed Child Development Centre and IDPA 500 participants from 33 countries Keynote lectures by self-advocates, advocacy panels, focus on a range of childhood disabilities and models of interventions
Third IDPA Congress (2019)	Manila, Philippines	Transdisciplinary Intervention: Ideas in Action	Hosted by the Philippine Society for Developmental and Behavioural Paediatrics and IDPA 660 participants from 20 countries Focused on innovative transdisciplinary models particularly relevant to LMICs where resources are low
Fourth IDPA Congress (2021)	Beirut, Lebanon (Virtual)	Nurturing Children in Crisis	Hosted by the Arab Network for Early Childhood Development and IDPA 936 participants from 79 countries Focused on impacts of the COVID-19 pandemic Addressed conflict, displacement and inequities affecting vulnerable children
Fifth IDPA Congress (2023)	Johannesburg, South Africa	Our Children Our Future	Hosted by Paediatric Neurology and Development Association of South Africa and IDPA 316 participants from 42 countries Continued focus on equity, disability and regional leadership
Sixth IDPA Congress (2025)	Guatemala City, Guatemala	Equity and Opportunities for All Children	Hosted by Wuqu Kawoq Maya Health Alliance and IDPA 455 participants from 40 countries Highlighted Indigenous approaches to early learning and caring for children with disabilities from Indigenous and minority communities. Expanded Global South collaboration and knowledge exchange Included scientific contributions in collaboration with the International Society for Social Paediatrics and Child Health

IDPA, International Developmental Pediatrics Association; LMICs, low-income and middle-income countries.

The 7^th^ IDPA Congress will be held in Baku, Azerbaijan, in 2027 and will be hosted by the Republican Paediatric Centre, with the theme ‘Supporting Every Child’s Unique Path’ recognising each child’s and family’s strengths, embracing neurodiversity and promoting evidence-based approaches that remain critically needed in the region^5^ and inclusive, family-centred and rights-based approaches.

Beyond its congresses, IDPA continues to support regional priorities related to disability, early childhood development, workforce capacity building and research. Through its international network, the organisation has facilitated the exchange of knowledge, mentorship and advocacy among professionals, trainees, researchers, families and self-advocates across diverse settings.

At a time when global discourse is calling for equitable approaches to child development and disability, IDPA represents an evolving model of collaboration grounded in LMIC leadership, scientific exchange and rights-based partnership. Sustaining and strengthening IDPA’s vision and equity-oriented practices remains critical to advancing global equity for children with developmental disabilities, their families and the professionals working to support children worldwide.
