# Supplementary material for: International Developmental Pediatrics Association: Global equity in child development and disability
Source: BMJ Paediatr Open. 2026 Aug 27;10(1):e004769. doi: 10.1136/bmjpo-2026-004769 (PMC13536136; doi:10.1136/bmjpo-2026-004769)
Supplement: Supplementary file 1 [file bmjpo-2026-004769.reviewer_comments.pdf]

PEER REVIEW HISTORY

BMJ Paediatrics Open publishes all reviews undertaken for accepted manuscripts. Reviewers are asked to complete a checklist review form and are provided with free text boxes to elaborate on their assessment. These free text comments are reproduced below.

ARTICLE DETAILS

**TITLE (PROVISIONAL)**	International Developmental Pediatrics Association (IDPA): Global equity in child development and disability.
**AUTHORS**	Ozalp Akın, Ezgi<br>Mustafayev, Revan<br>Srinivasan, Roopa<br>Sengupta, Koyeli<br>Pacifico, Ma. Rochelle<br>Charafeddine, Lama<br>Donald, Kirsten<br>Seyid Mammadova, Tarana

VERSION 1 – REVIEW

**REVIEWER NAME**	Gunnlaugsson, Geir
**REVIEWER AFFILIATION**	Faculty of Social and Human Sciences, University of Iceland
**REVIEWER CONFLICT OF INTEREST**	No Conflict of Interest
**AI DISCLOSURE**	No, I have not used AI
**DATE REVIEW RETURNED**	10-Jul-2026

**GENERAL COMMENTS**	The Editorial highlights the work of IDPA, which aims to promote equity in developmental paediatrics and child health worldwide. It rightly highlights the large number of disabled children in LMICs who lack adequate services, and the workforce that faces restricted access to training, mentorship, research and partnership opportunities. The Editorial, supported by a Table, illustrates the diverse themes in focus in the organisational work and the issues addressed at the biannual conferences, with evident strong participation from professionals around the world.<br><br>I have the following two suggestions for the authors:<br>On page 3 (out of 6), line 7, the sentence about IDPA conferences is missing the word "… has …"<br><br>Table. To harmonise with information from other conferences, the table lacks details on participants and on the number of countries.

**REVIEWER NAME**	Mercer, Raúl
**REVIEWER AFFILIATION**	Social Sciences and Health
**REVIEWER CONFLICT OF INTEREST**	No Conflict of Interest
**AI DISCLOSURE**	No, I have not used AI
**DATE REVIEW RETURNED**	12-Jul-2026

**GENERAL COMMENTS**	The article is a general comment that describes IDPA as a pediatric organization: its origin, role models, goals, and core values.<br>In terms of the specific contents:<br>Regarding the "Mentoring and Networking Dinner": is there any qualitative evaluation of the participants' opinions and the contribution of these activities to the development of their professional careers?<br>In Table 1 and regarding the 6th IDIPA Congress there is no mention to ISSOP (International Society for Social Pediatrics and Child Health) as a partner organization and its contribution to the development of the scientific program, contacting LAC stakeholders and contributing with many speakers. There is no mention of the number of participants and countries in this event.

VERSION 1 – AUTHOR RESPONSE

Responses to Reviewer 1
We thank Reviewer 1 for the constructive statements about the rationale and importance of this Viewpoint.
In response to the Reviewer's comments, our responses are as follows:
1. We thank the Reviewer for the careful reading. We carefully checked the cited page and line but could not identify the sentence referred to. In the current version, the sentence at that location refers to the upcoming 7th IDPA Congress and is intentionally written in the future tense; therefore, adding "has" would not be appropriate. We believe there may have been a discrepancy in the page or line reference, and no change has been made.
2. We have added the number of participants and the number of countries that were represented in the 6th IDPA Congress. This addition has been highlighted in yellow in Table 1.

Responses to Reviewer 2:

We thank Reviewer 2 for the comments and suggestions that have clarified and enriched our paper.
In response to the Reviewer's comments, our responses are as follows:
1.We thank the reviewer for this thoughtful suggestion. We agree that a formal qualitative evaluation of participant experiences and the contribution of the Mentoring and Mingling Dinner to their professional development would provide valuable evidence. The primary purpose of this event was to create a relaxed, informal environment that would encourage genuine conversations, mentorship, and networking across countries and career stages. To preserve the natural flow of these interactions, we did not conduct a structured evaluation during or immediately after the event. Nevertheless, based on informal feedback and conversations with participants, the dinner was perceived very positively and appeared to facilitate new professional connections, mentorship opportunities, research collaborations, and exchange of ideas.
As these impressions are empirical rather than systematically evaluated, we have revised the manuscript to avoid implying formally assessed outcomes. The addition has been highlighted in yellow in the last sentence of Page 2 and first sentence of Page 3.
We also recognize the formal evaluation of such activities as an important priority for future IDPA Congresses.

2. We thank the Reviewer for this helpful suggestion. Table 1 was intended to provide a concise overview of the main themes, host organizations and selected highlights of each congress rather than a comprehensive list of all collaborating organizations and scientific contributors. Across six congresses; numerous international, regional, and local organizations including WHO, UNICEF, professional societies, universities, and civil society organizations, have contributed to the scientific

programs in different capacities. We agree that ISSOP made a valuable contribution to the scientific program of the 6th IDPA Congress and we have added this in Table 1 (highlighted in yellow) while maintaining the concise format of the table.
